# Comparative Efficacy of Direct Oral Anticoagulants and Low-Molecular-Weight Heparin in Cancer-Associated Thromboembolism: A Systematic Review and Meta-Analysis

**DOI:** 10.7759/cureus.41071

**Published:** 2023-06-28

**Authors:** Tirath Patel, Taha Nadeem, Usman Shahbaz, Fatima Tanveer, Muneeb Ahsan, Usman Saeed, Abdullah Ahmed, Vinesh Kumar, Syed M Ibne Ali Jaffari, Mohammad U Zaman, Satesh Kumar, Mahima Khatri, Giustino Varrassi, Prasanthi Vanga

**Affiliations:** 1 Medicine, American University of Antigua, Saint John, ATG; 2 Medicine, Allama Iqbal Medical College, Lahore, PAK; 3 Internal Medicine, Jinnah Hospital, Lahore, PAK; 4 Medicine, Chandka Medical College, Larkana, PAK; 5 Medicine and Surgery, Shalamar Medical and Dental College, Lahore, PAK; 6 Medicine, Bacha Khan Medical College, Mardan, PAK; 7 Medicine and Surgery, Shaheed Mohtarma Benazir Bhutto Medical College, Karachi, PAK; 8 Medicine and Surgery, Dow University of Health Sciences, Karachi, PAK; 9 Pain Medicine, Paolo Procacci Foundation, Rome, ITA; 10 Medicine, Konaseema Institute of Medical Sciences and Research Institute, Amalapuram, IND

**Keywords:** cancer, venous thromboembolism, lmwhs, low molecular weight heparins, direct oral anticoagulants, doacs

## Abstract

Patients diagnosed with cancer often experience an abnormal occurrence of venous thromboembolism (VTE) and its related complications. In order to evaluate the safety and effectiveness of both treatment approaches, we conducted a comprehensive systematic review and meta-analysis within the realm of cancer-associated thromboembolism. A thorough search was conducted across PubMed, the Cochrane Library, and Embase databases to find studies comparing direct oral anticoagulants (DOACs) with low molecular weight heparins (LMWHs) for the treatment of VTE in patients with malignancy. The analyses utilized the random-effects model. This meta-analysis included 11 studies. The results showed that DOACs were associated with a significantly reduced risk of VTE recurrence (RR: 0.67; 95% CI: 0.55, 0.81, p<0.0001; I2: 0%) and deep vein thrombosis (DVT) (RR: 0.63; 95% CI: 0.46, 0.86, p<0.0001; I2: 0%) compared to LMWHs. However, there was no significant difference in the risk of pulmonary embolism (PE) (RR: 0.76; 95% CI: 0.54, 1.06, p=0.11; I2: 11%) between the two groups. The use of DOACs was also associated with a non-significant increase in the risk of major bleeding events (RR: 1.23; 95% CI: 0.85, 1.78, p: 0.26; I2: 49%), while clinically relevant non-major bleeding (CRNMB) was significantly higher with DOACs (RR: 1.92; 95% CI: 1.11, 3.30, p: 0.02; I2: 81%). Secondary outcomes, such as survival rates and fatal PE, did not show significant differences between the two treatment groups. Our analysis indicates that direct oral anticoagulants exhibit a substantial decrease in the occurrence of VTE recurrence, deep vein thrombosis, and pulmonary embolism when compared to low molecular weight heparin in cancer-associated thromboembolism. However, it should be noted that DOACs carry a higher risk of CRNMB. Based on these findings, DOACs are recommended as a superior therapeutic option for managing cancer-associated thromboembolism compared to LMWH.

## Introduction and background

Venous thromboembolism (VTE), comprising deep-vein thrombosis (DVT) and pulmonary embolism (PE), presents a considerable health concern, manifesting an approximate yearly occurrence of one to two cases per 1,000 individuals in the overall populace [1]. Nevertheless, cancer patients exhibit an imbalanced prevalence of VTE and its associated complications. In contrast to non-cancer patients, the likelihood of experiencing VTE among cancer patients is believed to be amplified by a factor ranging from 4 to 6.5 [2]. VTE is additionally recognized as a prominent cause of mortality among cancer patients [3]. Consequently, a hypothesis has emerged suggesting that malignancy induces a state of heightened blood clotting susceptibility in patients with cancer [4].

The potential occurrence of cancer-associated VTE varies depending on the specific type of cancer, with a heightened risk often observed in individuals afflicted with metastatic disease [4,5]. Various established risk-scoring models have been developed to assess the likelihood of cancer-associated VTE. These models encompass a range of factors derived from clinical characteristics (such as tumor type and body mass index), laboratory measurements (including hemoglobin levels and platelet counts), and biomarkers (such as soluble P-selectin and D-dimer) [5]. Managing VTE in patients with cancer can be challenging due to cancer-related complications, including an augmented vulnerability to bleeding and potential interactions between chemotherapy and other medications. In contrast to non-cancer patients, who may be recommended long-term oral therapy with vitamin K antagonists (VKAs) following initial heparin treatment for VTE, VKAs are not advised for cancer-associated VTE [6]. Patients diagnosed with cancer exhibit a risk of anticoagulant-related bleeding up to six times higher than those without cancer, with a three- to four-fold elevated likelihood of VTE recurrence even after undergoing VKA treatment [6].

Among individuals with cancer-associated VTE, the utilization of low molecular weight heparin (LMWH), such as enoxaparin or dalteparin, demonstrates comparable or reduced rates of VTE recurrence and bleeding compared to VKAs [7]. Notably, LMWH does not necessitate gastrointestinal absorption and exhibits minimal interactions with chemotherapy. As a result, both clinical guidelines and practical recommendations endorse LMWH as the preferred initial therapy for short-term and long-term management of cancer-associated VTE [7]. Direct factor Xa inhibitors (such as rivaroxaban, apixaban, edoxaban, and betrixaban) offer several advantages over VKAs, including a fixed-dose regimen, predictable anticoagulant effects, and the absence of routine laboratory monitoring requirements. Furthermore, unlike LMWH, direct oral anticoagulants (DOACs) eliminate the need for long-term subcutaneous injections and can be taken orally, alleviating potential barriers associated with LMWH treatment [7,8]. Regarding treating acute VTE, DOACs have demonstrated comparable efficacy to VKAs as a class, albeit with a slightly higher propensity for minor bleeding events. Until recently, limited clinical studies directly compared the safety and effectiveness of DOACs and LMWH in managing cancer-related VTE [8].

Previous clinical guidelines, predating 2018, provided limited recommendations regarding using DOACs for managing cancer-associated VTE. This scarcity of guidance stemmed from an insufficient amount of available data at the time of their publication [8,9]. Until recently, the information available to inform the utilization of DOACs in patients with cancer-associated VTE consisted primarily of mid-level data derived from pivotal phase III clinical trials of each DOAC, primarily through secondary analyses [9]. Considering the available evidence, DOACs appear viable and often preferred options for VTE treatment in cancer patients, mainly when there is a concern regarding potential interactions with chemotherapy drugs or a heightened risk of bleeding [10]. Given the scarcity of comprehensive data and the inconsistencies observed in comparative studies, we undertook a systematic review and meta-analysis to assess both treatment modalities' safety and efficacy profiles in cancer-associated thromboembolism.

## Review

Methodology 

In terms of methodology, this meta-analysis adheres to the guidelines outlined by the Preferred Reporting Items for Systematic Review and Meta-analysis (PRISMA) [11], thereby ensuring a comprehensive and rigorous approach to the study's design and execution.

*Data Sources and Search Strategy* 

A thorough and exhaustive exploration of the existing literature was undertaken to locate pertinent studies for this investigation. Prominent electronic databases such as PubMed, Embase, and the Cochrane Library were meticulously queried until May 2023. The search strategy involved employing a combination of specific terms such as "direct oral anticoagulants," "DOACs," "low molecular weight heparin," "LMWH," "malignancy-associated venous thromboembolism," "cancer," "meta-analysis," and other associated phrases. Furthermore, a manual examination of reference lists from relevant articles and conference proceedings was performed to identify any additional relevant studies. Adjustments were made to the population, intervention, comparison, and outcome (PICO) methodology to best suit the objectives of this study. Notably, patients with a history of neoplasm-associated hypercoagulopathy were included in the study population. The titles and abstracts of potentially eligible studies were evaluated independently by three researchers (TP, TN, and FT).

Inclusion and Exclusion Criteria 

Inclusion criteria: The selection of studies was conducted by applying a set of rigorous inclusion criteria: RCTs and observational studies (cohort or case-control studies) were considered. The focus of the studies had to be on evaluating the effectiveness of DOACs or LMWH in treating VTE associated with malignancy. The selected studies were required to report on appropriate outcome measures, including VTE recurrence, bleeding events, survival rates, and adverse events. The studies had to be published in English to ensure accessibility and comprehensibility. The studies exclusively included adult patients who had been diagnosed with malignancy-associated VTE. These meticulous criteria were applied with the utmost attention to detail, ensuring valuable and relevant research was included in the analysis and synthesis process.

Exclusion criteria: The following categories were meticulously excluded from consideration: research involving animals, review articles, editorials, case reports, case series, and conference abstracts. Moreover, studies lacking a control group, duplicate publications, and datasets with overlapping information were carefully eliminated from our purview. These rigorous measures were undertaken to uphold our study's integrity and guarantee our findings' reliability.

Data Extraction 

To ensure meticulousness and accuracy, two impartial reviewers diligently extracted pertinent data from the designated studies utilizing a standardized data extraction form. The collected information encompassed several crucial aspects. First, the study characteristics were documented, including the author, year of publication, study design, sample size, and follow-up duration. Second, patient characteristics were recorded, encompassing age, sex, type of cancer, cancer stage, and comorbidities. Information was also gathered regarding the intervention employed, specifically the type of DOACs utilized. Furthermore, the type of LMWH used for comparison purposes was also documented. Moreover, primary outcomes of interest involved the recurrence of VTE, major bleeding events, and the incidence of DVT, PE, and clinically relevant non-major bleeding (CRNMB). Secondary outcomes included survival rates, mortality due to any cause, incidental PE, fatal PE, and minor bleeding events. In the event of any discrepancies or diverging opinions between the reviewers, they were resolved through constructive discussion or by involving a third reviewer.

*Quality Assessment of the Included Studies* 

The assessment of the studies included in this research was conducted with great care and attention to ensure the reliability and credibility of the findings. To evaluate the quality and potential biases, we employed established and suitable methodologies, including the Cochrane Risk of Bias Tool [12] for RCTs and the Newcastle-Ottawa Scale [13] for observational studies. In this comprehensive evaluation, we scrutinized several critical aspects: randomization, allocation concealment, blinding, completeness of follow-up, and selective reporting.

Data Analysis and Synthesis 

The statistical analysis for this meta-analysis was performed using Review Manager version 5.4.1 (The Nordic Cochrane Centre in collaboration with The Cochrane Collaboration, Denmark). Additionally, STATA 16 (STATA Corporation, College Station, Texas) was utilized in the analysis. Only comparative studies were considered for inclusion in this analysis. The findings were presented through forest plots, which depict the combined effect of relative risks (RRs) for dichotomous outcomes and weighted mean differences (WMDs) for continuous outcomes. The generic-inverse variance with a random-effects model was employed to ensure the accuracy of the results.

To determine the significance of the results, the threshold for statistical significance was set at a p-value of less than 0.05. In order to evaluate the possibility of publication bias, funnel plots were generated for each primary outcome. The interpretation of these plots was further verified by conducting Beggs's test specifically for primary outcomes that were reported by 10 or more studies. Furthermore, Higgin's I2 test was employed to assess the level of heterogeneity, which was subsequently categorized as low, moderate, or high.

In cases where high heterogeneity (greater than 75%) [14] was detected, a sensitivity analysis was conducted by systematically excluding one study at a time. This allowed for the identification of individual study contributions to the overall findings. Moreover, a subgroup analysis was performed based on the type of LMWH used. This analysis aimed to ensure the efficacy and safety of DOACs compared to the widely used types of LMWH drugs.

Importantly, as the information utilized in this study was gathered and consolidated from previously conducted clinical trials where participants had already provided informed consent, ethical approval from a research ethics committee was not required by the research team.

Results 

Eligible Studies 

**Figure 1:**
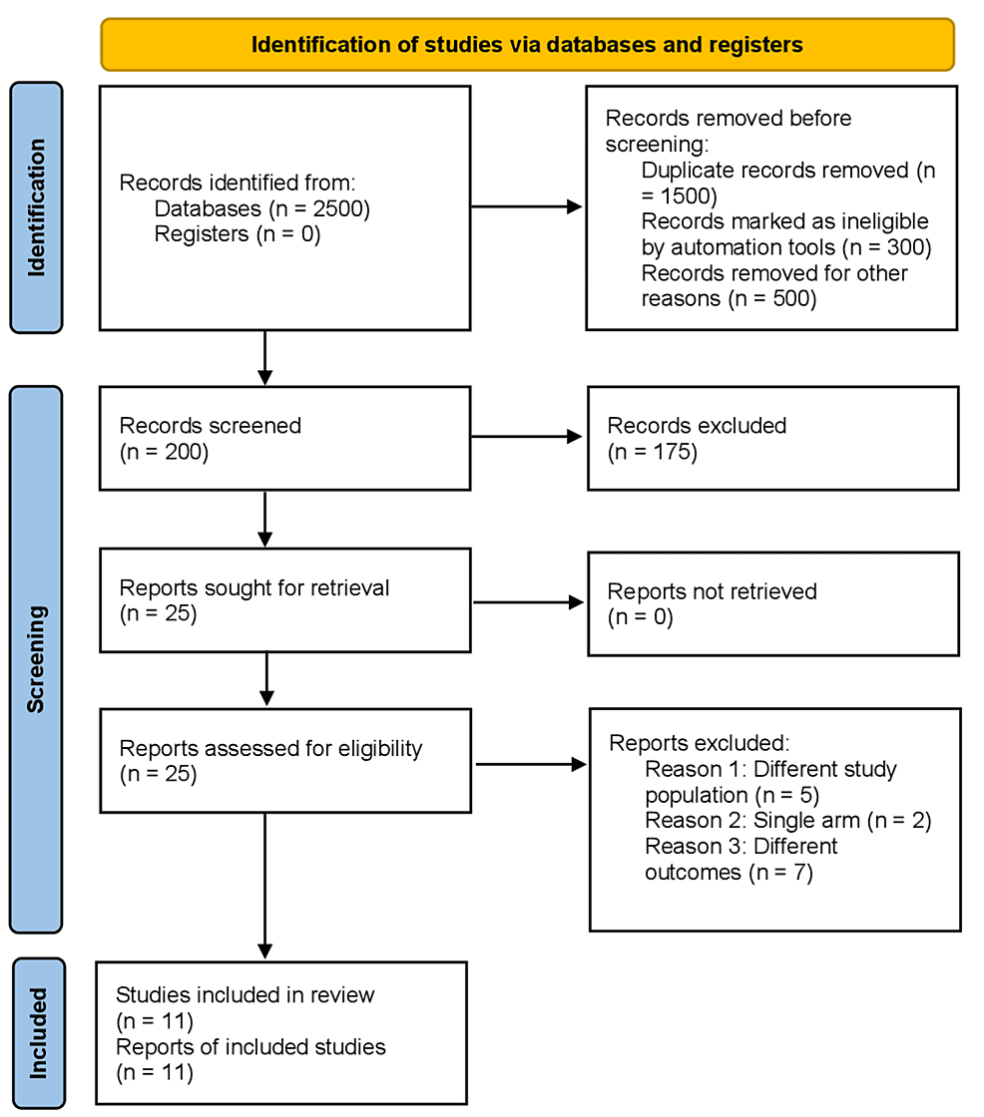
PRISMA diagram illustrating the selection process for inclusion of studies in the meta-analysis The Preferred Reporting Items for Systematic Reviews and Meta-Analyses (PRISMA) diagram showcases the step-by-step procedure employed to identify and select studies for inclusion in the meta-analysis. Initially, 2500 articles were identified through a literature review. After removing duplicates and carefully evaluating titles and abstracts, 11 studies that met the inclusion criteria were chosen.

The initial outcome of the literature review consisted of 2500 articles. Subsequently, 11 studies were identified for inclusion by removing duplicates and carefully assessing titles and abstracts. This meta-analysis specifically focused on comparative studies. The PRISMA diagram in Figure 1 illustrates the comprehensive search strategy employed. Out of the 11 studies [15-25], three were observational in nature [19,20,24], while the remaining eight were double-blind randomized controlled trials. All of the RCTs included in this analysis were conducted across multiple centers. The mean duration of follow-up for these studies was seven months. It should be noted that the collection of articles selected for this analysis covers the period from 2010 to 2022.

Baseline Characteristics of the Included Patients 

This comprehensive analysis incorporated 8,051 participants, with 4,008 individuals (49.7%) assigned to the DOACs group and 4,043 (50.2%) assigned to the LMWH group. The study population mainly comprised females, accounting for 4,930 participants (61.2%) out of 8,051, while the remaining 3,121 participants (38.7%) were male. The average age of the participants was 63.4 ± 12.1 years. At the beginning of the study, the majority of patients, precisely 5,219 participants (64.8%), had deep vein thrombosis, whereas 2,083 participants (25.8%) had a pulmonary embolism, and 647 participants (8%) had both DVT and PE. Most patients had early or locally advanced solid cancer, with a subset having metastatic or hematological malignancies. Among the patients receiving treatment for their primary cancer, the majority were undergoing chemotherapy. Further details regarding the baseline characteristics of the included patients can be found in Table 1.

**Table 1:** Baseline characteristics of the included participants DOACs: direct oral anticoagulants, LMWH: low molecular weight heparin.

Study and year	Total no. of participants	No. of patients		Types of treatment		Male gender no.		Age (mean ± SD)		Early/locally advanced disease		Metastatic disease	
		DOACs	LMWH	DOACs	LMWH	DOACs	LMWH	DOACs	LMWH	DOACs	LMWH	DOACs	LMWH
Planquette et al. [15]	158	74	84	Rivaroxaban	Dalteparin	37	40	68.6± 2.75	70.7± 2.96	16	20	53	62
Agnelli et al. [16]	1155	576	579	Apixaban	Dalteparin	292	275	67.4± 2.75	68± 2.96	N/A	N/A	386	345
Oliveira et al. [17]	228	114	114	Rivaroxaban	Enoxaparin	N/A	N/A	54± 11.8	56± 11.4	5	2	109	112
Young et al. [18]	406	203	203	Rivaroxaban	Dalteparin	116	98	67± 12	67± 9.8	51	79	118	118
Kim et al. [19]	174	69	105	Rivaroxaban	Dalteparin	32	56	67± 2.4	59± 2.6	11	13	58	92
Recio-Boiles et al. [20]	106	66	40	Rivaroxaban	Enoxaparin	41	18	68 ± 7.4	66 7.4	N/A	N/A	N/A	N/A
Raskob et al. [21]	1043	522	524	Edoxapan	Dalteparin	277	263	64.3 ±11	63.7± 11.7	513	511	274	280
EINSTEIN Investigators [22]	3449	1731	1718	Rivaroxaban	Enoxaparin	993	967	55.5± 16.4	56.4± 16.3	118	89	N/A	N/A
Kim et al. [23]	90	44	46	Rivaroxaban	Dalteparin	25	23	64± 7	63± 10	44	46	38	33
Chen et al. [24]	1109	559	580	Dabigatran, rivaroxaban, apixaban, and edoxaban	Enoxaparin	246	285	67.7± 13.3	64.6± 12.4	240	220	211	235
Mokadem et al. [25]	100	50	50	Apixaban	Enoxaparin	20	22	61.2± 11.2	59.9± 9.71	N/A	N/A	N/A	N/A

*Quality Assessment and Publication Bias* 

**Figure 2:**
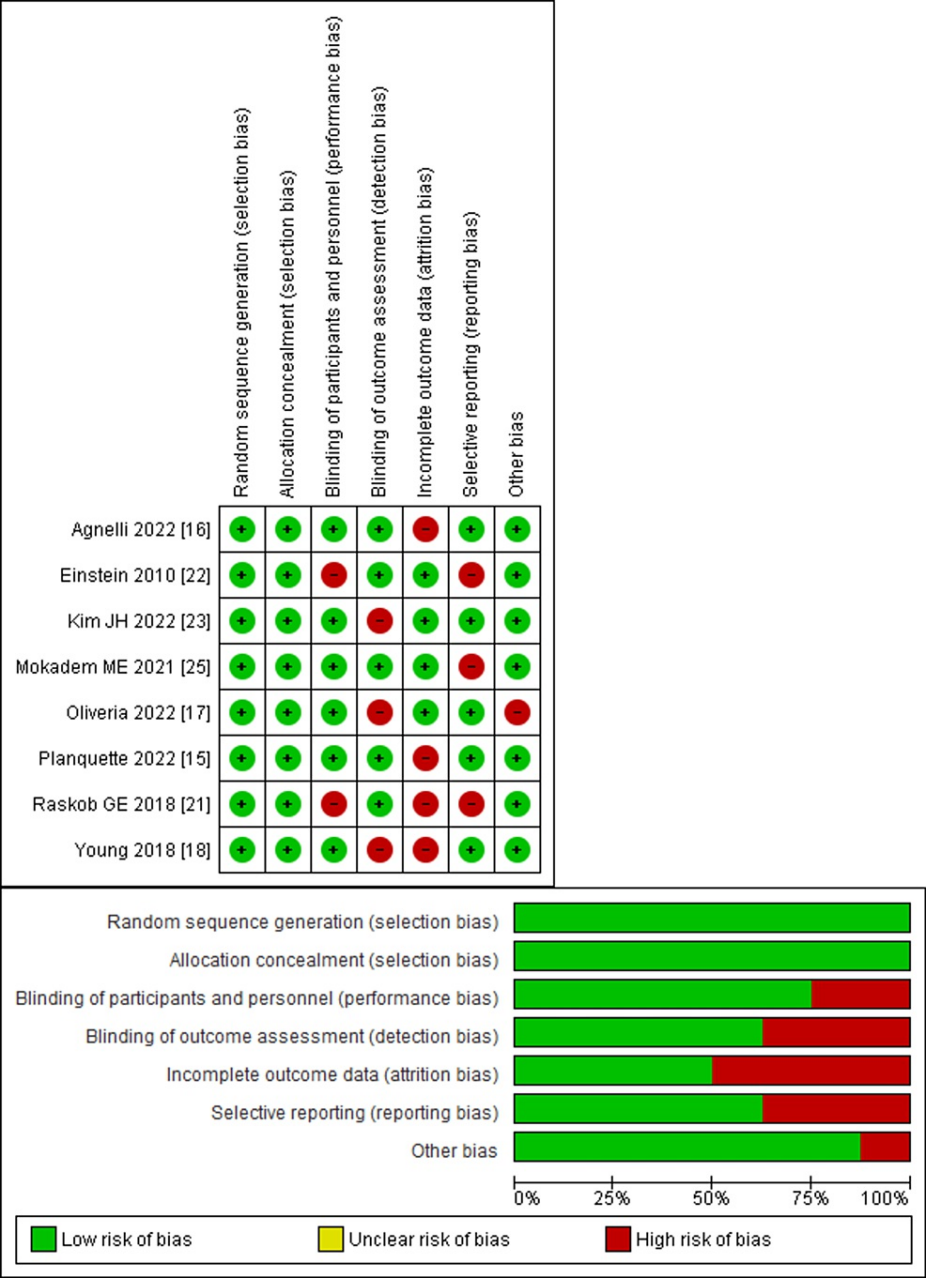
Representation of the Cochrane risk of bias assessment for randomized controlled trials The figure highlights the presence of trials with moderate-to-high quality, indicating a reliable and robust study design. Source: references [15-18,21-23,25].

**Figure 3:**
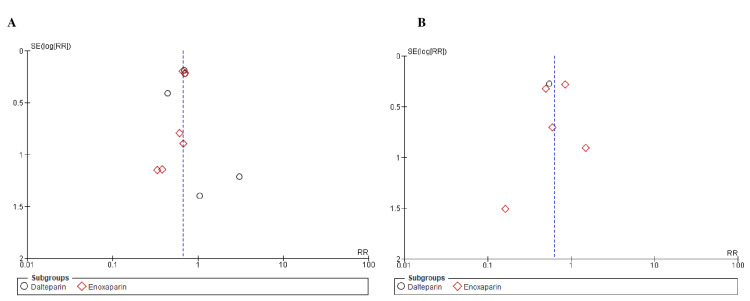
Funnel plots of primary outcomes (A) Venous thromboembolism recurrence and (B) deep vein thrombosis. RR: risk ratio, SE: standard error.

**Figure 4:**
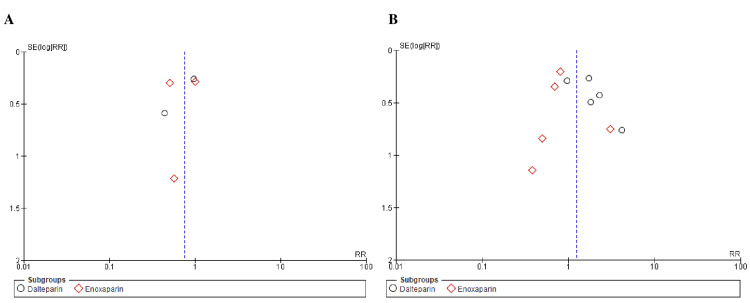
Funnel plots of primary outcomes (A) pulmonary embolism and (B) major bleeding. RR: risk ratio, SE: standard error.

**Figure 5:**
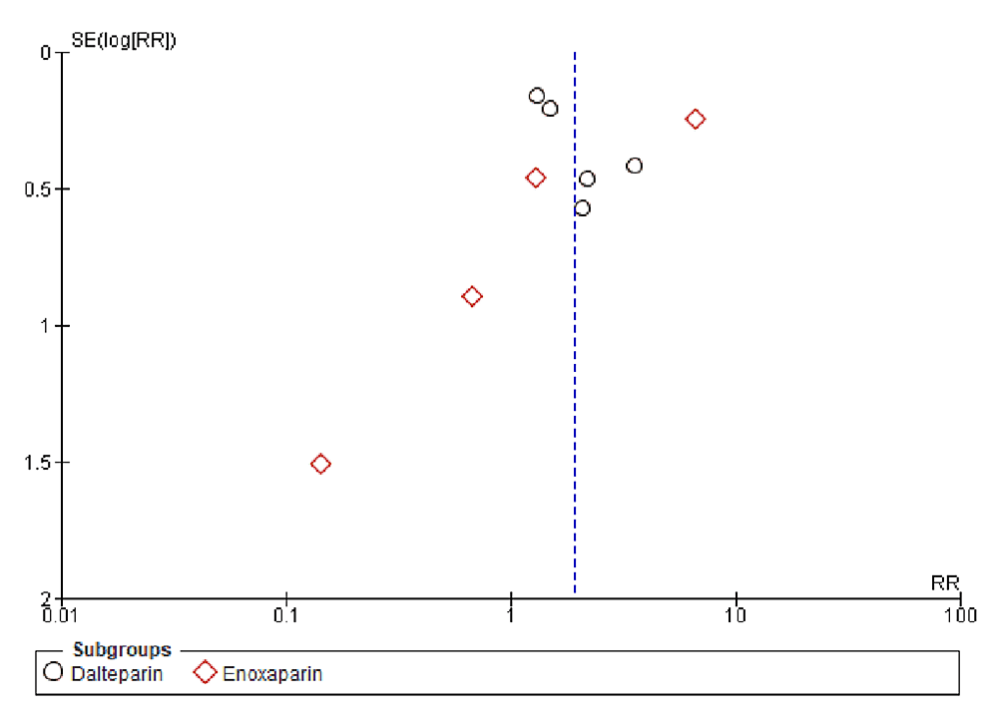
Funnel plots of primary outcomes: clinically relevant non-major bleeding RR: risk ratio, SE: standard error.

The New Castle-Ottawa scale, employed to evaluate the quality of observational studies, indicated a minimal risk of bias, as shown in Table 2. When using the Cochrane risk of bias tool to assess RCTs, trials of moderate-to-high quality were found, as demonstrated by Figure 2. Notably, the results remained unaffected by any potential publication bias, as shown by the informative funnel plots of the primary outcomes, as shown in Figures 3-5, and confirmed by the comprehensive findings of Begg's test, as shown in Table 3.

**Table 2:** Newcastle-Ottawa Scale for quality assessment of observational studies Good quality studies are characterized by three or four stars in the selection domain, along with one or two stars in the comparability domain, and two or three stars in the outcome/exposure domain. These ratings indicate that the study has strong selection criteria, moderate comparability to other studies, and favorable outcomes or exposure measures. Fair quality studies are defined by two stars in the selection domain, combined with one or two stars in the comparability domain, and two or three stars in the outcome/exposure domain. This classification suggests that the study has acceptable selection criteria, moderate comparability, and satisfactory outcomes or exposure measures. On the other hand, poor quality studies receive a rating of zero or one star in the selection domain, zero stars in the comparability domain, or zero or one star in the outcome/exposure domain. These ratings indicate weak selection criteria, lack of comparability to other studies, or unfavorable outcomes or exposure measure.

Study	Selection				Comparability	Outcomes			Total
	Comparability of cohorts on the basis of the design or analysis controlled for confounders	Selection of the non-exposed cohort	Ascertainment of exposure	Demonstration that outcome of interest was not present at the start of the study	Comparability of cohorts on the basis of the design or analysis controlled for confounders	Assessment of outcome	Was follow-up long enough for outcomes to occur	Adequacy of follow-up of cohorts	
Kim et al. [19]	*	*	*	*	**	*	*	*	*********
Boiles et al. [20]	*	*	*	*	*	*	*	*	********
Chen et al. [24]	*	*	*	*	**	*	*	*	*********

**Table 3:** Begg's test VTE: venous thromboembolism.

Outcomes	Begg's test
VTE recurrence	1.466
Major bleeding	0.1524

*Primary Outcomes* 

The primary outcomes analyzed encompassed the rate of recurrent venous thromboembolism, deep vein thrombosis, pulmonary embolism, major bleeding events, and clinically relevant non-major bleeding.

**Figure 6:**
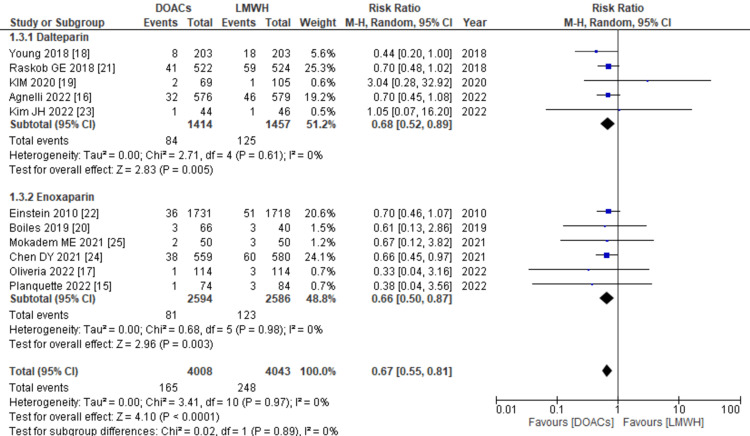
Forest plot of VTE recurrence The results of the comparative analysis, indicate a significant reduction in the risk of VTE recurrence associated with the use of DOACs compared to LMWH. The findings further revealed that regardless of whether dalteparin or enoxaparin was utilized as the control drug, DOACs consistently exhibited a lower risk of VTE recurrence. VTE: venous thromboembolism, DOACs: direct oral anticoagulants, LMWHs: low molecular weight heparins, RR: relative risk, CI: confidence interval, M-H: Mantel Hansel. Source: references [15-25].

Recurrence of venous thromboembolism: All 11 studies provided information on the rate of VTE recurrence. When the data were combined, it revealed that the use of DOACs for treatment was linked to a significantly reduced risk of VTE recurrence compared to LMWH (RR: 0.67; 95% CI: 0.55, 0.81, p<0.0001; I2: 0%), as shown in Figure 6. Additionally, the subgroup analysis indicated that regardless of whether dalteparin or enoxaparin was used as the control drug, the risk of VTE recurrence was consistently lower with DOACs.

**Figure 7:**
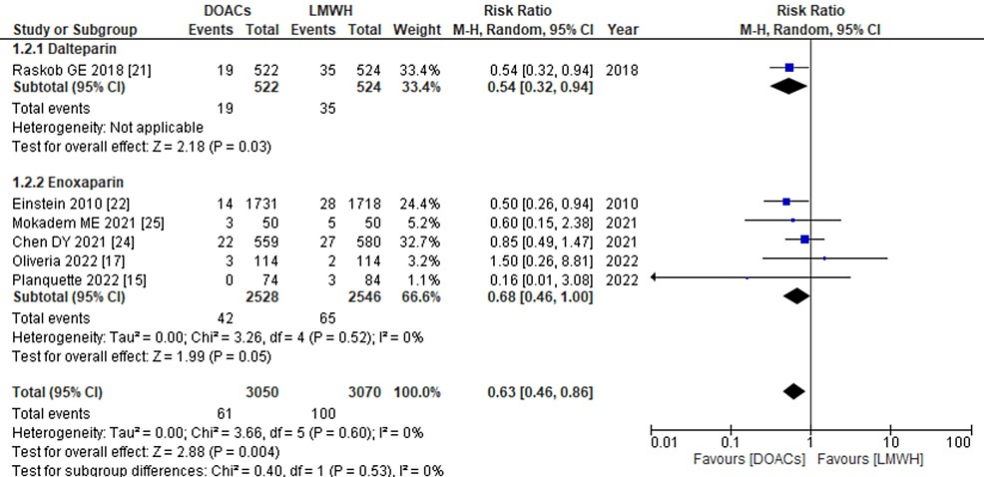
Forest plot of incidence of DVT The figure illustrates the comparison between the use of DOACs and LMWH, revealing a significantly lower risk of DVT with DOACs. Notably when dalteparin was employed as the control arm, the reduced risk of DVT was particularly pronounced. DVT: deep vein thrombosis, DOACs: direct oral anticoagulants, LMWHs: low molecular weight heparins, RR: relative risk, CI: confidence interval, M-H: Mantel Hansel. Source: references [15,17,21,22,24,25].

Deep vein thrombosis: Data on the occurrence of DVT during the follow-up period were provided by six out of 11 studies. When the data were pooled and analyzed, it was found that the use of DOACs for treatment was significantly associated with a lower risk of DVT compared to LMWH (RR: 0.63; 95% CI: 0.46, 0.86, p: <0.0001; I2: 0%), as depicted in Figure 7. This reduced risk was particularly notable when dalteparin was used as the control arm (RR: 0.54; 95% CI: 0.32, 0.94, p=0.03), in comparison to when enoxaparin was used as the control arm (RR: 0.68; 95% CI: 0.46, 1.00, p=0.05; I2: 0%).

**Figure 8:**
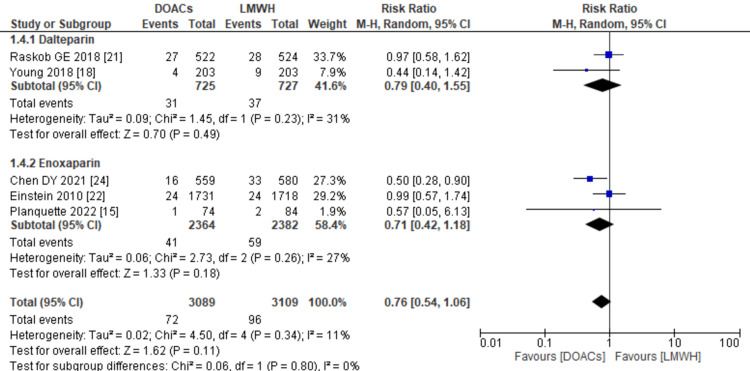
Forest plot of the risk of PE This figure illustrates that the use of DOACs was associated with a non-significant decrease in the risk of PE. PE: pulmonary embolism, DOACs: direct oral anticoagulants, LMWHs: low molecular weight heparins, RR: relative risk, CI: confidence interval, M-H: Mantel Hansel. Source: references [15,18,21,22,24].

Pulmonary embolism: Incidence data on pulmonary embolism was provided by five of the 11 studies. The combined analysis indicated that the use of DOACs for treatment was linked to a non-significant decrease in the risk of PE compared to LMWHs (RR: 0.76; 95% CI: 0.54, 1.06, p=0.11; I2: 11%), as depicted in Figure 8. Subgroup analysis further demonstrated that the reduced risk associated with DOACs remained consistent regardless of whether dalteparin or enoxaparin was used in the control arm.

**Figure 9:**
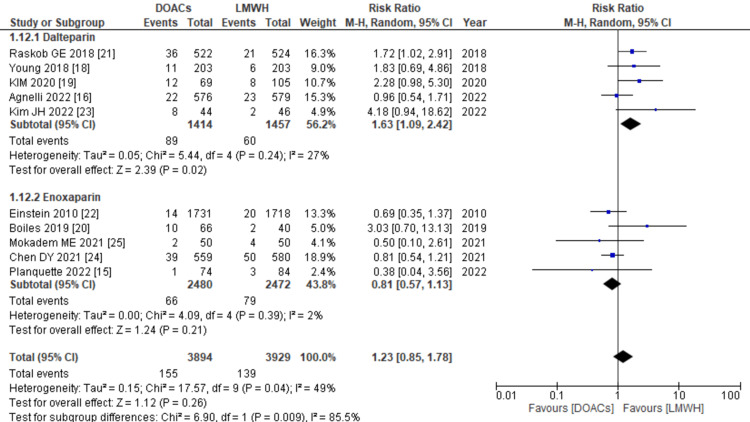
Forest plot of the risk of major bleeding The figure depicts that the use of DOACs was associated with a non-significant increase in the risk of major bleeding. However, subgroup analysis revealed that when enoxaparin was used as the control arm, treatment with DOACs was linked to a non-significant decrease in the risk of major bleeding. In contrast, when dalteparin was used as the control drug, the use of DOACs significantly increased the risk of major bleeding. DOACs: direct oral anticoagulants, LMWHs: low molecular weight heparins, RR: relative risk, CI: confidence interval, M-H: Mantel Hansel. Source: references [15,16,18-25].

Major bleeding: The incidence of major bleeding was reported in 10 of the 11 studies. The pooled analysis revealed that the use of DOACs for treatment was linked to a non-significant increase in the risk of major bleeding compared to LMWHs (RR: 1.23; 95% CI: 0.85, 1.78, p=0.26; I2: 49%), as depicted in Figure 9. However, upon conducting subgroup analysis, it was observed that when enoxaparin was used as the control arm, treatment with DOACs was associated with a non-significant decrease in the risk of major bleeding (RR: 0.81; 95% CI: 0.57, 1.13, p=0.21; I2: 2%). Conversely, when dalteparin was used as the control drug, DOACs significantly increased the risk of major bleeding (RR: 1.63; 95% CI: 1.09, 2.42, p=0.02; I2: 27%).

**Figure 10:**
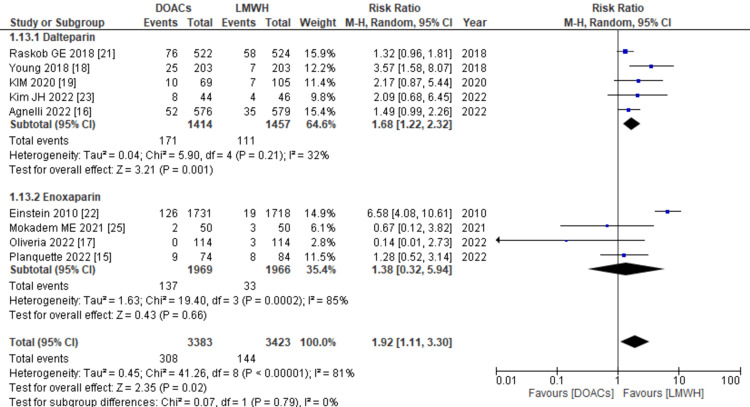
Forest plot of the risk of CRNMB The figure illustrates the association between the use of DOACs for treatment and an increased risk of CRNMB compared to LMWHs. Subgroup analysis indicated that this increased risk remained consistent with DOACs, regardless of whether enoxaparin or dalteparin was used as the control drug. CRNMB: clinically relevant non-major bleeding, DOACs: direct oral anticoagulants, LMWHs: low molecular weight heparins, RR: relative risk, CI: confidence interval, M-H: Mantel Hansel. Source: references [15-19,21-23,25].

Clinically relevant non-major bleeding: Incidence data on CRNMB was provided by nine out of 11 studies. The combined analysis demonstrated a significant association between the use of DOACs for treatment and an increased risk of CRNMB compared to LMWHs (RR: 1.92; 95% CI: 1.11, 3.30, p=0.02; I2: 81%), as illustrated in Figure 10. However, upon conducting subgroup analysis, it was observed that the increased risk remained consistent with DOACs regardless of whether enoxaparin or dalteparin was used as the control drug. Given the substantial heterogeneity among the included studies, a leave-one-out sensitivity analysis was performed. Notably, after excluding the study conducted by EINSTEIN Investigators [22], a significant reduction in heterogeneity was observed (RR: 1.56; 95% CI: 1.16, 2.11, p=0.004; I2: 25%).

Secondary Outcomes 

The secondary outcomes assessed in this meta-analysis encompassed survival rates, fatal PE, incidental PE, minor bleeding, clinically overt bleeding with a decrease in hemoglobin (Hb) exceeding 2 g/dl over 24 hours, clinically overt bleeding necessitating transfusion of more than two packed red cells, clinically overt bleeding leading to death, and death from any cause. The pooled analysis demonstrated a significant reduction in the risk of clinically overt bleeding leading to death with DOAC treatment compared to LMWHs. Conversely, the analysis indicated a non-significant decrease in the risk of incidental PE, clinically overt bleeding, a decrease in Hb of more than 2 g/dl over 24 hours, and death from any cause associated with DOACs. Furthermore, DOAC treatment was associated with a non-significantly increased risk of minor bleeding and clinically overt bleeding leading to transfusion of more than two packed red cells. However, no significant differences were observed between the two groups in terms of survival or fatal PE. Given the substantial heterogeneity observed in the outcomes of survival and death from any cause, a leave-one-out analysis was conducted for each outcome.

The analysis revealed that excluding the studies conducted by Agnelli et al. [16] and EINSTEIN Investigators [22] one by one significantly reduced the heterogeneity in the survival outcome. Similarly, excluding the study conducted by Chen et al. [24] significantly reduced heterogeneity in the death from any cause outcome. The values of the analysis of secondary outcomes can be found in Table 4.

**Table 4:** Secondary outcomes RR: risk ratio, CI: confidence interval, I2: heterogeneity, PE: pulmonary embolism, Hb: hemoglobin.

Outcome	Effect size (RR)	CI	p-value	I2
Survival	1.01	0.92, 1.12	0.80	87%
Incidental PE	0.69	0.32, 1.49	0.35	22%
Fatal PE	1.00	0.17, 5.75	1.00	0%
Minor bleeding	1.10	0.92, 1.31	0.28	0%
Clinically overt and decrease in Hb of >2 g/dl over 24 hours	0.90	0.46, 1.75	0.75	0%
Clinically overt and transfusion of >2 packed red cells	2.06	0.93, 4.55	0.07	52%
Clinically overt bleeding leading to death	0.33	0.12, 0.91	0.03	0%
Death from any cause	0.80	0.56, 1.13	0.21	87%

Discussion

Although the correlation between thrombosis and individuals with cancer is widely recognized, it is imperative to acknowledge that various subgroups within the cancer population exhibit distinct risk distributions for thromboembolic events. Numerous anti-cancer medications, including antiangiogenic agents, multitargeted tyrosine kinase inhibitors, combinations of immunomodulatory drugs, and potentially even immunotherapy protocols, heighten the likelihood of VTE. This risk has persisted despite the advent of newer anti-cancer therapies [26]. VTE exerts adverse effects on cancer patients in two significant manners: directly, by elevating short-term mortality rates, hospitalizations, and emergency room visits; and indirectly, by associating with inferior outcomes, such as reduced survival rates and diminished quality of life [26]. To investigate the risk factors associated with different subtypes of DOACs in cancer patients, we performed subgroup analyses focusing on medications such as dabigatran, rivaroxaban, apixaban, and edoxaban.

In this comprehensive meta-analysis, comprising 11 studies and involving a total of 8,051 patients, we conducted a comparative assessment of the effectiveness and safety of DOACs in contrast to LMWHs for managing venous thromboembolism associated with cancer. DOACs demonstrated notably superior efficacy in preventing the recurrence of VTE when compared to LMWHs. No superiority regarding recurrent VTE has been demonstrated for any DOAC, neither in individual randomized controlled trials (RCTs) nor in the combined findings of previous meta-analyses. The enhanced effectiveness of DOACs in reducing the risk of VTE recurrence compared to LMWHs could be partially attributed to improved compliance with orally administered medication instead of subcutaneous injections. This might be evident in the lower rate of premature treatment discontinuation observed in patients receiving oral factor Xa inhibitors. In the study by O'Connell et al., similar results were found. VTE recurrence rates at six months were 4% for the rivaroxaban group and 11% for the dalteparin group, with an HR of 0.43 and a 95% CI ranging from 0.19 to 0.99. Each treatment arm had two cases of symptomatic PE, and one fatal PE occurred in each. The primary tumor site significantly influenced the occurrence of VTE recurrence. Stomach or pancreas cancer patients faced a more than fivefold higher risk of recurrence (HR = 5.55; 95% CI = 1.97-15.66), while lung, lymphoma, gynecologic, or bladder cancer patients had a more than twofold increased risk (HR = 2.69; 95% CI = 1.11-6.53) [27].

Furthermore, in comparison to LMWH, DOACs exhibited a significantly lower incidence of DVT in therapeutic applications [5]. Recent randomized, double-blind, placebo-controlled studies have provided information on the use of DOACs in ambulatory cancer patients. The Apixaban for the Prevention of Venous Thromboembolism in High-Risk Ambulatory Cancer Patients (AVERT) study focused on patients with a Khorana score of two initiating a new course of chemotherapy. It demonstrated that apixaban-treated patients had a significantly lower occurrence of objectively documented major VTE, specifically proximal DVT or pulmonary embolism, than those treated with a placebo [5]. Advanced-stage cancers are commonly associated with a higher prevalence of VTE, which often presents as a pulmonary embolism, either with or without concurrent DVT in the lower limbs [28]. In three studies by Mulder et al., which specifically enrolled patients with proximal DVT or PE, the primary analysis indicated that the occurrence of recurrent VTE was slightly lower in the group receiving DOACs compared to the group receiving LMWH. However, this difference was not statistically significant (RR = 0.68, with moderate-quality evidence). Compared to LMWHs, the utilization of DOACs for therapy did not significantly increase the risk of severe bleeding, based on the overall analysis, advocating against using DOACs for people with a significant risk of bleeding. The rates of combined bleeding (major and non-major) at 6 and 12 months were comparable between DOACs (16.7% and 20%, respectively) and LMWHs (15.4% and 17.9%), as reported in a study by Ross et al. The average time interval before bleeding occurred was one month. Although significant bleeding events were slightly more frequent in individuals receiving DOACs (13% versus 11% for LMWHs), clinically relevant minor hemorrhage was similar in both groups, with rates of 7.3% for LMWHs and 6.7% for DOACs [28].

Most participants in the clinical trials received systemic cancer treatment. Medications that either inhibit or promote P-glycoprotein or cytochrome P-450 3A4, including certain chemotherapy drugs, tyrosine kinase inhibitors, tamoxifen, and immunomodulating substances like dexamethasone, have the potential to alter the plasma concentrations of DOACs. However, we could not assess these potential interactions due to the absence of data regarding this specific patient category. Further research is necessary to understand the pharmacokinetic properties of DOACs when used concomitantly with cancer medications [28]. The studies included in this meta-analysis encompassed a diverse population of cancer patients, with a consistent and substantial proportion (ranging from 53% to 65%) having metastatic disease. It is crucial to note that the four individual trials comprising this meta-analysis exhibited notable discrepancies. In the Hokusai VTE Cancer, SELECT-D, and CARAVAGGIO trials, the risk of severe bleeding varied between 4% and 6% in the DOAC group and between 3% and 4% in the LMWH group. ADAM-VTE, excluded from the primary analysis, reported no bleeding episodes in the DOAC group and only two cases (1.4%) in the LMWH group [5]. This discrepancy could be attributed to factors such as lower mortality risk in ADAM-VTE or the enrollment of patients with upper-extremity DVT and splanchnic vein thrombosis, which were not included in the other studies. However, the secondary analysis results incorporated the ADAM-VTE study and did not demonstrate significant differences [28].

Our study has some limitations. First, including a diverse patient population with different types of cancer may introduce variations in the findings. Second, the unavailability of patient-level data hindered the assessment of comparative outcomes between DOACs and LMWH based on primary cancer or VTE types. Third, the data synthesis was solely based on published results, and the analysis did not include patient-level data. Finally, there is a need for a more specific investigation into the effects of different DOACs on particular types of cancer. Future studies should prioritize examining the impact of various DOACs on cancer types to assist clinicians in making informed treatment decisions for patients with different types of cancer and VTE.

## Conclusions

Our systematic review and meta-analysis findings indicate that DOACs demonstrate a significant reduction in the risk of VTE recurrence, DVT, and PE compared to LMWH in the context of cancer-associated thromboembolism. However, it is essential to note that DOACs are associated with an increased risk of CRNMB. Based on the evidence, DOACs present a superior therapeutic option over LMWH for managing cancer-associated thromboembolism.
